# Reactive Oxygen Species-Based Biomaterials for Regenerative Medicine and Tissue Engineering Applications

**DOI:** 10.3389/fbioe.2021.821288

**Published:** 2021-12-23

**Authors:** Muhammad Shafiq, Yujie Chen, Rashida Hashim, Chuanglong He, Xiumei Mo, Xiaojun Zhou

**Affiliations:** ^1^ Shanghai Engineering Research Center of Nano-Biomaterials and Regenerative Medicine, College of Chemistry, Chemical Engineering and Biotechnology, Donghua University, Shanghai, China; ^2^ Department of Biotechnology, Faculty of Life Science, University of Central Punjab (UCP), Lahore, Pakistan; ^3^ Department of Chemistry, Faculty of Science, Quaid-i-Azam University (QAU), Islamabad, Pakistan

**Keywords:** reactive oxygen species, oxidative stress, inflammatory response, regenerative medicine, tissue engineering

## Abstract

Reactive oxygen species (ROS), acting as essential mediators in biological system, play important roles in the physiologic and pathologic processes, including cellular signal transductions and cell homeostasis interference. Aberrant expression of ROS in tissue microenvironment can be caused by the internal/external stimuli and tissue injury, which may leads to an elevated level of oxidative stress, inflammatory response, and cellular damage as well as disruption in the tissue repair process. To prevent the formation of excess ROS around the injury site, advanced biomaterials can be remodeled or instructed to release their payloads in an injury microenvironment-responsive fashion to regulate the elevated levels of the ROS, which may also help downregulate the oxidative stress and promote tissue regeneration. A multitude of scaffolds and bioactive cues have been reported to promote the regeneration of damaged tissues based on the scavenging of free radicals and reactive species that confer high protection to the cellular activity and tissue function. In this review, we outline the underlying mechanism of ROS generation in the tissue microenvironment and present a comprehensive review of ROS-scavenging biomaterials for regenerative medicine and tissue engineering applications, including soft tissues regeneration, bone and cartilage repair as well as wound healing. Additionally, we highlight the strategies for the regulation of ROS by scaffold design and processing technology. Taken together, developing ROS-based biomaterials may not only help develop advanced platforms for improving injury microenvironment but also accelerate tissue regeneration.

## Introduction

Reactive oxygen species (ROS) are highly reactive ions or free radicals, such as superoxide (O_2_^−^), hydrogen peroxide (H_2_O_2_), hydroxyal radicals (·OH), hypochlorite ion (ClO^−^), singlet oxygen (^1^O_2_) and so on. The ROS play a significant role in homeostasis and physiological functions, which are precisely modulated by their amount, duration, and localization. Both, a lack or an excess of ROS may cause different types of diseases, including autoimmune disease, cardiovascular disease, and neurodegenerative disease (Saravanakumar et al., 2017; Hameister et al., 2020). While the wound healing and tissue repair processes are governed by the different phases of inflammation, the excessive inflammation may exacerbate oxidative stress and ROS. Most of the tissues, including skin, bone, soft tissues (muscle, ligament, fascia nerves, fibrous tissues, fat, blood vessels, synovial tissues and so on) are susceptible to oxidative stress. For instance, ROS act as signaling molecules in managing multiple endothelial cells (ECs) and smooth muscle cells pathways, including their proliferation, migration, and apoptosis. However, the excessive ROS accumulated at the injury site can induce strong inflammatory response to interfere with the tissue repair process. The ROS level under pathological conditions can exceed 500 μm in inflammatory tissues, which is much higher than that in the normal tissues (1–15 μm). While on the one hand, excessive ROS at the injury site can perturb tissue repair through overlying signaling pathway and oxidative stress, on the other hand, the excessive ROS can lead to the damage of the biological molecules and disturbance of the immune system. The ROS-mediated altered cellular behaviour may lead to inflammatory signaling resulting into leukocytes and platelets activation as well as their recruitment. Moreover, the implantation of biomaterials has been found to elevate oxidative stress as well as recruit polymorphonuclear cells and monocytes, which may further increase inflammatory response. Therefore, a thorough understanding of ROS and oxidative stress will help understand biomaterials-host interactions and interrogate the injury microenvironment.

**FIGURE 1:**
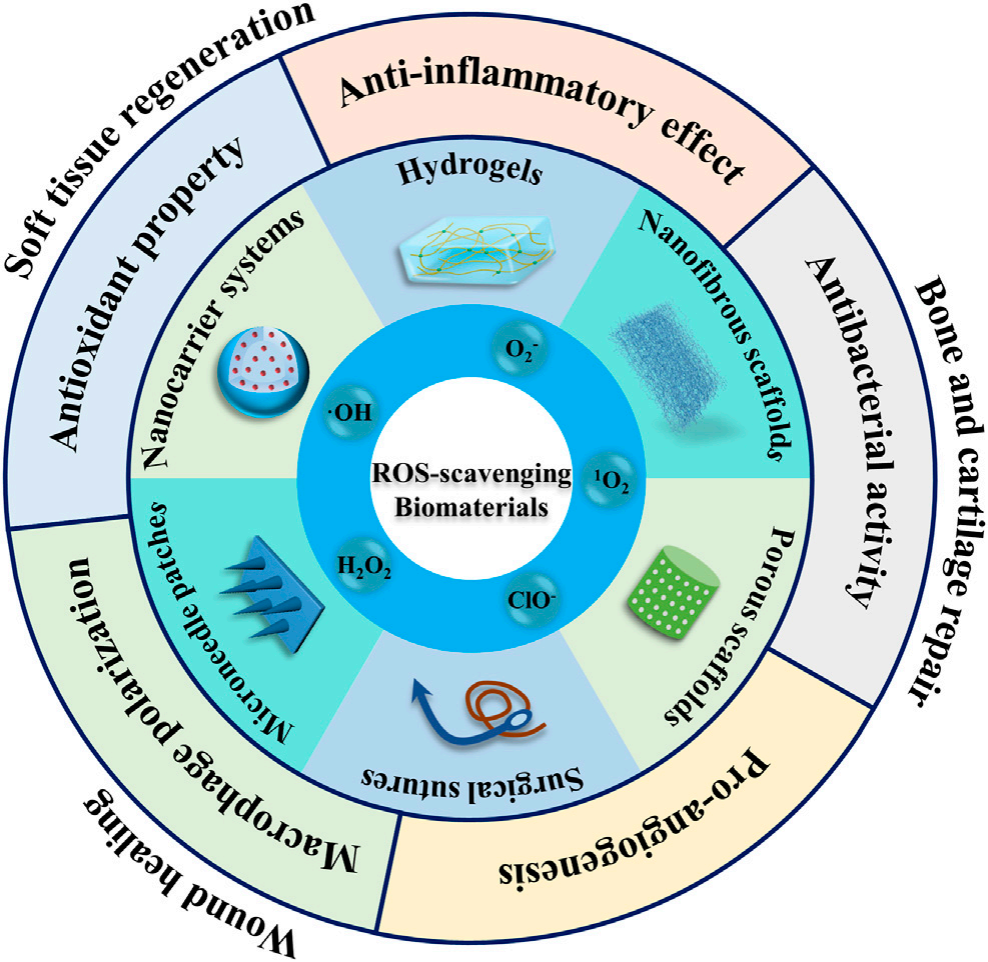
Schematic illustration of the application of reactive oxygen species (ROS)-responsive biomaterials in multiple fields. ROS-regulating biomaterials have been fabricated into different shapes and structures, such as surgical sutures, porous scaffolds, nanofibrous scaffolds, hydrogels, nanocarriers and microneedles.

Since systematic ROS scavengers can interfere with the several physiological processes, it is imperative to develop localized ROS scavengers. While substantial research has been carried out on the use of ROS-sensitive materials for drug delivery applications and cancer therapy, ROS-responsive biomaterials which can simultaneously release their payloads and regulate ROS level in the injured tissues, while simultaneously impact tissue repair process have received only a little attention (Lee et al., 2013; Saravanakumar et al., 2017). Nonetheless, these instructive biomaterials may possess several functions, such as the regulation of the injury microenvironment by the alleviation of the oxidative stress or ROS, which may help modulate inflammatory response and inflammatory signaling mediated cellular processes, harness oxidative stress and/or ROS as microenvironmental cues to afford drug/growth factor release, and induce the degradation of implanted scaffolds. We have discussed the role of ROS-sensitive biomaterials for the regeneration of soft tissues (nerves, heart, and muscles), osteochondral tissues (bone and cartilage) and wound healing (Figure 1). As oxidative stress and ROS are central to almost all types of tissues, these can be an important design criterion in biomaterials engineering and worthy of further investigations.

### Regulation of ROS for Soft Tissues Regeneration

ROS pose debilitating threats for various types of diseases of soft tissues, such as atherosclerosis, ischemia, diabetes, and myocardial infarction/reperfusion injuries. Excessive ROS species also impair tissue regeneration. Particularly, excessive ROS can cause inflammation and fibrosis, which can further mount injury burden. Consequently, timely resolution of ROS holds great promise. Wagner et al., designed polymers with an inherent ability to scavenge ROS by conjugating 4-Amino-2,2,6,6-tetramethylpiperidine-1-oxyl (TEMPO) to the thermo-responsive hydrogels (Zhu et al., 2018). In a myocardial reperfusion/injury model, these hydrogels reduced apoptosis and preserved left ventricular (LV) function. Similarly, Bai et al., have designed ROS-degradable hydrogels to lower the degeneration of the intervertebral disk. It was delineated that the scaffolds-mediated ROS suppression led to the controlled release of rapamycin, which also resulted into macrophages polarization by increasing the number of M2 macrophages while depleting the number of M1 macrophages (Bai et al., 2020). Liu et al., leveraged hyper-branched poly (*β*-amino esters) to scavenge ROS, which ameliorated infarcted heart conditions (Wang et al., 2019).

To scavenge ROS, a myriad of strategies have been used, including the use of radical scavenging polymers, ROS-responsive crosslinkers, biologics, or therapeutics (Zhao et al., 2020; Chen Z. et al., 2021). Gu et al., exploited poly (ester amide) (PEA) as an antioxidant and blended it with poly (acrylic acid) (PAA), which not only scavenged free radicals but also promoted cell growth and viability as well as angiogenesis (Zhang J. et al., 2021). The ROS scavenging ability of these hydrogels emanated from the arginine release. Likewise, Duvall et al. have exploited poly (propylene sulphide) (PPS) to scavenge ROS and release anti-oxidative and anti-inflammatory curcumin (Poole et al., 2015). Since PPS is a hydrophobic polymer, it can efficiently improve drug efficiency and can trigger the drug release in a ROS-responsive manner by transforming into more hydrophilic poly (propylene sulfoxide) and poly (propylene sulfone) polymers. Consequently, curcumin-encapsulating PPS microparticles considerably decreased the ROS level in activated macrophages and oxidative stress induced cell death *in vitro*. Besides, these biomaterials increased angiogenesis in a hind-limb ischemia model. While the incorporation of antioxidants and radical scavengers can afford the reduction of the oxidative stress and ROS-related cell dysfunction, the immobilization or encapsulation of these moieties into scaffolds provides only a marginal benefit. To circumvent this limitation, inherently anti-oxidative polymers have been synthesized (Van Lith et al., 2014; Shiekh et al., 2018). Kumar et al. synthesized ascorbic acid incorporated polyurethane (Shiekh et al., 2018). Since ascorbic acid was incorporated into the main chain of the polymer, the localized and sustained presence of the antioxidant successfully reduced the oxidative stress in H9C2 cardiomyocytes and ROS-induced cell death, which may have great promise for the treatment of diseased conditions with increased oxidative stress, such as cardiovascular diseases, chronic wounds, and myocardial infarction. Liu et al. have designed poly (vinyl alcohol) (PVA) based hydrogels crosslinked via ROS-responsive linkers and systematically studied their ROS scavenging ability *in vitro* in human umbilical vein endothelial cells (HUVECs) and *in vivo* in lipopolysaccharide (LPS)-induced infection model and diabetes/infection model (Zhao et al., 2020). Importantly, these hydrogels, not only scavenged ROS as indicated by the lower expression of the H_2_O_2_ and OH but also induced the release of mupirocin and granulocyte-macrophage colony-stimulating factor (GM-CSF), which increased antibacterial effect and macrophages polarization toward M2 phenotypes, respectively. By using a similar approach, Shan et al. designed ROS-responsive PVA based hydrogels, which not only modulated inflammation in injured skeletal muscles but also promoted the survival of mesenchymal stem cells (MSCs) under oxidative environment as well as induced myogenesis and macrophages polarization toward M2 phenotypes (Shan et al., 2021). Cheng et al. further exploited these ROS-responsive PVA polymers to afford an epicardial patch, which sustainably and controllably released fibroblast growth factor-2 (FGF-2) *in vitro* in response to the H_2_O_2_ as well as improved its retention in the pericardial cavity *in vivo* via overproduced ROS following ischemia-reperfusion injury in mice. More importantly, these scaffolds also reduced cardiac fibrosis and promoted neo-myogenesis (Li Z. et al., 2021). Likewise, Martin et al. synthesized ROS-sensitive poly (thioketal)urethane (PTK-UR), which degraded in a cell-mediated manner upon an increase in the ROS and induced tissue regeneration after subcutaneous implantation (Martin et al., 2021). Gao et al. designed ROS cleavable hyperbranched polymers and leveraged them along with methacrylated hyaluronic acid (HAMA) to afford ultraviolet (UV)-crosslinkable hydrogels (Ding et al., 2020). Importantly, while ROS-cleavable linker can scavenge ROS, catalase could generate oxygen from the decomposition of H_2_O_2_, making this combinatorial strategy capable of alleviating hypoxia and rejuvenating oxygen, and enhancing their suitability for the treatment of various pathological hallmarks, including myocardial infarction (MI). Consequently, these hydrogels promoted cell viability under oxidative stress as well as reduced infarct size and promoted angiogenesis in an MI model.

Nitric oxide (NO) has several implications for maintaining vascular hemostasis, which can be activated by cells expressing endothelial NO synthase, inducible NO synthase, and neuronal NO synthase. The NO has been delivered to promote neo-vessel formation in blood vessels. However, given to the short half-life of NO, the therapeutic level cannot be maintained. Besides, elevated levels of ROS rapidly react with the NO to reduce its bioavailability. Nagasaki et al. designed poly (arginine) based NO-releasing and ROS-responsive hydrogels, which not only afforded the NO release by the degradation of arginine moieties but also suppressed ROS in a redox-sensitive manner (Vong et al., 2018). Hydrogels simultaneously eluting NO and scavenging ROS significantly reduced infarct size as well as improved blood vessel formation than that of their counterparts which only released NO or scavenged ROS in MI model in mice.

To date, different types of strategies have been adopted to scavenge ROS and alleviate free radicals, such as the application of oxidant scavengers and engineered enzymes. However, the low bioavailability of therapeutic compounds and their tendency to become oxidants at high doses limit their applications (Table 1). Consequently, a sustained and localized delivery of antioxidants may improve the clinical benefits of lowering the oxidative stress. Jain et al. leveraged cerium oxide nanoparticles (nCe) to alleviate oxidative stress and scavenge ROS (Jain et al., 2021). Nanofibrous poly (caprolactone)/gelatin (PCL/Gel) scaffolds containing nCe reduced ROS level in H_2_O_2_-induced cardiomyocytes as well as suppressed agonist-induced cardiac hypertrophy. Therefore, further research is warranted to afford nanomaterials-based ROS scavengers, which may have broad applications. Similarly, other members of the antioxidants, such as fullerenol have been leveraged to improve stem cell function, especially in the ROS microenvironment. The ROS can be adhered to the electron deficient positions of fullerenol nanoparticles; alleviating the oxidative stress. Once delivered along with extracellular matrix (ECM)-mimetic alginate hydrogels, fullerenol nanoparticles were reported to scavenge ROS, alleviate oxidative stress, and improve stem cell function, such as survival, retention, and engraftment by activating mitogen-activated protein kinase (MAPK) signaling pathways (i.e. ERK, p38, and JNK, etc), which collectively translated into enhanced clinical outcome, including angiogenesis and cardiac functional recovery (Hao et al., 2017). Wang et al. leveraged hydrogen sulphide (H_2_S)-releasing electrospun PCL fibers by encapsulating N-(benzoylthio)benzamides derivatives (NSHD), which can induce H_2_S release triggered by biological thiols such as cysteine and glutathione (GSH), which prevail in the biological system (Feng et al., 2015). The H_2_S releasing nanofibrous scaffolds promoted the survival and proliferation of myoblasts and fibroblasts by reducing the oxidative stress.

**TABLE 1:** Reactive oxygen species-regulating biomaterials for tissue regeneration applications.

Bioactive cue/scaffold	Animal model	Key findings	References
Rapamycin-loaded scaffolds	Intervertebral disk	Macrophages polarization toward M2 phenotypes increased while ROS level decreased	Bai et al. (2020)
ROS-cleavable polymers, catalase, 4-amino-TEMPO based HA hydrogels	Myocardial infarction	ROS level and infarct size decreased while cell viability, cardiac function and angiogenesis increased	Ding et al. (2020), Wang et al. (2019), Zhu et al. (2018)
Nitric oxide releasing poly (arginine) hydrogels		Nitric oxide release and angiogenesis increased while ROS level and infarct size decreased	Vong et al. (2018)
Curcumin-loaded poly (propylene sulphide) nanoparticles	Hind-limb ischemia or reperfusion	ROS level, oxidative stress, and cell apoptosis decreased while limb regeneration increased	Poole et al. (2015)
Ascorbic acid loaded polyurethane		Oxidative stress and cell apoptosis decreased	Shiekh et al. (2018)
FGF-2-loaded cardiac patch		FGF-2 release and neo-myogenesis increased while cardiac fibrosis decreased	Li Z. et al. (2021)
ROS-scavenging PLGA hydrogels	Bone/cartilage	Inflammation decreased while glycosaminoglycans and collagen increased	Wu et al. (2021)
BMP-2-loaded PTK-based coatings		BMP-2 delivery and bone regeneration increased	Martin et al. (2021)
PEA-PAA hydrogels	Skin injury	Cell growth, cell viability, wound healing, and arginine release increased	Zhang J. et al. (2021)
Mupirocin and GM-CSF-loaded PVA scaffolds		ROS level decreased while M2 macrophages and wound healing increased	Zhao et al. (2020)
Tannic acid, curcumin or *Spirulina* extract loaded chitin hydrogels or PCL nanofibers		Antibacterial activity, wound healing, angiogenesis, and anti-inflammatory activity increased while oxidative stress decreased	Jung et al. (2016), He et al. (2020), Ma et al. (2020), Yang et al. (2021)
Gallic acid loaded hydrogels or sutures		Oxidative damage decreased while cell viability, neovascularization, and wound repair increased	Le Thi et al. (2020), Zhu et al. (2021)
EGF-loaded PEG hydrogels		ROS level and scar formation decreased while EGF release and skin repair increased	An et al. (2022)
MnO_2_-loaded HA hydrogels		ROS level decreased while oxygen release and angiogenesis increased	Xiong et al. (2021)
Curcumin and Zn^2+^-loaded PLLA scaffolds		ROS level and inflammatory response decreased while Zn^2+^ and epithelialization increased	Wang Y. et al. (2020)
PNA nanogel-loaded PLLA nanofibers		Cell adhesion and proliferation increased while ROS level decreased	Zhang J. et al. (2021)
Ce6, Mg^2+^, and EGCG loaded chitosan NPs		Mg^2+^ release, skin repair, and antibacterial activity increased while ROS level decreased	Hu et al. (2019)
SDF-1α-loaded PPADT		SDF-1α release, skin repair, and BMSCs homing increased	Tang et al. (2015)
Clindamycin-loaded PVA microneedle patch		Bacterial growth decreased while drug penetration increased	Zhang et al. (2018)
Ceria-loaded PCL scaffold	*In vitro* studies	ROS level and cardiac hypertrophy decreased	Jain et al. (2021)
H_2_S-releasing scaffolds		H_2_S, cell survival and proliferation increased	Feng et al. (2015)

EGF, epidermal growth factor; PLLA, poly(l-lactic acid); EGCG, epigallocatechin-3-gallate.

### Regulation of ROS for Bone and Cartilage Tissue Repair

ROS signaling is also enhanced during bone and cartilage degeneration, which is considerably exacerbated with an increase in inflammation, such as in osteoarthritis (OA). The chondrocytes are quiescent under normal conditions, and they reside in a hypoxic environment due to the lack of blood vessels in cartilaginous tissues. Upon the disruption of the mitochondrial function, chondrocytes become activated, which upregulates the expression of the intracellular ROS, further disrupting homeostasis and increasing OA (Blanco et al., 2011). Furthermore, a myriad of literatures have established age-mediated exacerbation of the oxidative stress causing an imbalance in ROS production and antioxidant capacities (Carlo and Loeser, 2003).

A multitude of antioxidants and free radical scavengers, such as phenols (e.g., vitamin E), ubiquitin (e.g., vitamin Q), flavonoids (e.g., curcumin, quercetin), thiols (e.g., glutathione, thioredoxin) (Grover and Samson, 2016; Chin and Ima-Nirwana, 2018), xanthan gum and alginate (Chen et al., 2017), dopamine melanin (Zhong et al., 2019), fullerene and fullerol (Pei et al., 2019), chondroitin sulfate (CS) and hyaluronic acid (Chen et al., 2017; Chen Y. et al., 2021) have been demonstrated to reduce the oxidative stress, thus delaying cartilage degeneration. These supplements upregulate the expression of antioxidants (e.g., superoxide dismutase, glutathione, catalase and nuclear factor erythroid 2-related factor 2 and lead to the downregulation of ROS (Li et al., 2016; Marchev et al., 2017). For example, theaflavins (TFs), the primary active polyphenols in black tea, have been extensively investigated for their antioxidative abilities as well as antiviral and anticancer activities (Maron et al., 2003). Li et al. explored the protective effect of TFs on chondrocytes and reported a decrease in the ROS level and cell apoptosis (Li and Zheng, 2019). In addition, CS, a naturally occurring glycosaminoglycans (GAGs) which constitutes soft tissues has been widely explored for its anti-inflammatory properties (Abe et al., 2016). Woo et al. reported a reduction in the secretion of inflammatory cytokines and ROS in LPS-treated murine RAW 264.7 macrophages after treatment with skate cartilage CS (Woo et al., 2020).

However, systematically or orally administered antioxidants exhibit poor retention at the injury site, which necessitates their spatiotemporal presentation. Liang et al. explored the antioxidative potential of PCL-grafted lignin in H_2_O_2_-stimulated human chondrocytes and rabbit OA model, which was plausibly mediated by an autophagic mechanism (Liang et al., 2020). Pei et al. leveraged water-soluble polyhydroxylated fullerene C60 (fullerol) nanoparticles to scavenge ROS, which significantly reduced LPS-induced NO production and pro-inflammatory gene expression *in vitro* as well as lowered inflammation in an OA model (Pei et al., 2019). Yet another study explored the antioxidative potential of alpha-tocopheryl succinate (α-TOS) and tumor necrosis factor alpha (TNF-α) siRNA co-loaded poly (amidoamine) dendrimer-entrapped gold nanoparticles (Au DENPs) (Li et al., 2020). The *α*-TOS enhanced the antioxidant capacity of macrophages. The *in vivo* studies further established the downregulation of the inflammatory cytokines in OA. Li et al. developed tannic acid/strontium (TA/Sr^2+^) coated silk/graphene oxide-based meniscal scaffolds to modulate inflammatory response and reduce ROS in the OA model, which displayed anti-inflammatory and ROS scavenging abilities as well as downregulated the expression of inflammatory factors, including interleukin-6 (IL-6), interleukin-8 (IL-8), and matrix metalloproteinases (MMPs) in rat knee tissues (Li Y. et al., 2021). Similarly, ionic liquids (ILs) have been incorporated into bacterial nanocellulose (BC) based membranes, which exhibited anti-inflammatory and antioxidant properties (Sintra et al., 2015). We have recently developed metal organic frameworks (MOFs) decorated mesoporous polydopamine nanoparticles and conjugated collagen-II-targeting peptide for OA therapy (Xue et al., 2021). Rapamycin and bilirubin were loaded into the MOFs and polydopamine shells to modulate inflammatory response and activate autophagic mechanism, respectively. These nanoparticles delayed cartilage degeneration as well as modulated the inflammatory response and protected chondrocytes. The inflammatory factors, such as interleukin-1 (IL-1), IL-6, Interleukin-1*β* (IL-1*β*), and TNF-α as well as ROS species are widely expressed after cartilage degeneration, which provoke the inflammatory reaction. Consequently, Gao et al. designed hybrid scaffolds consisting of poly (l-lactide-co-glycolide) (PLGA) and ROS-scavenging HAMA hydrogels containing ROS-sensitive hyperbranched polymers with thioketal linkages, which not only modulated ROS but also regulated inflammation and promoted hyaline cartilage regeneration (Wu et al., 2021).

Similarly, different types of biomaterials have been designed to regulate ROS level for bone tissue regeneration. Huang et al. designed aniline tetramer (AT) and glycine ethyl ester co-substituted polyorganophosphazene (PATGP) based scaffolds (Huang et al., 2020). The AT moieties possessed antioxidative properties and phosphazene induced bone regeneration, which exhibited ROS-scavenging effect in a cranial defect model. Lee et al. fabricated bone morphogenetic protein-2 (BMP-2) loaded PCL/TA scaffolds, which displayed antioxidative and anti-inflammatory properties by scavenging ROS in H_2_O_2_-pretreated MC3T3-E1 cells (Lee et al., 2018). Similarly, Zhou et al. exploited polypyrrole-polydopamine (PPy-PDA) coating on hydroxyapatite scaffolds, which exhibited ROS scavenging ability both *in vivo* and *in vitro* (Zhou et al., 2019). The composite porous scaffold provided excellent bone regeneration through the synergistic effects of electroactivity, cellular affinity, and antioxidant activity of PPy-PDA nanoparticles and osteogenic induction of hydroxyapatite NPs. Wang et al. prepared a series of novel chemically modified N-polyphenol substituted chitosan derivatives, which displayed antioxidative activity as well as promoted the differentiation of MSCs (Wang J. et al., 2020). Hammond et al. further exploited poly (thioketals) (PTK) to afford layer-by-layer coatings to deliver BMP-2 for calvarial defect regeneration in a ROS mediated manner (Martin et al., 2021). Cell-mediated increase in the ROS can cleave the PTK-based LBL coatings, which can then release BMP-2 to promote bone repair and may have broad applications to leverage therapeutics delivery in a stimuli-responsive manner. Similarly, Chen et al. synthesized chitosan nitrogen-doped carbon dots (N-CDs) to scavenge ROS (Chen et al., 2020). The N-CDs effectively inhibited osteoclast formation and bone resorption *in vitro*. In addition, the *in vivo* administration of N-CDs protected mice from LPS-induced cranial bone destruction and breast cancer cell-induced tibial bone loss.

## Regulation of ROS for Wound Healing

Wound healing is a complex biological process that occurs in various tissues/organs of human body, consisting of multiple phases including hemostasis, inflammation, proliferation and remodeling (Wu et al., 2019). It has been well-established that wounds express high ROS level as compared to normal tissues. The excessive amount of ROS generated at the wound sites aggravate oxidative stress, which eventually leads to dysfunction in the cellular machinery, including DNA/RNA damage, protein dysfunction and apoptosis. As a result, the elevated production of ROS can perturb the wound healing. Therefore, different types of ROS-responsive biomaterials have been developed and implemented for wound repair, including hydrogels, nanofibrous materials, nanoparticulate drug delivery systems, surgical sutures and microneedle patches by the direct or indirect management of local ROS production to regulate wound healing process (Figure 1 and Table 1).

Hydrogel dressings, a kind of biomaterials that can provide a moist environment due to their three-dimensional (3D) porous network with high water content, are considered as ideal candidates for promoting wound healing (Yin et al., 2021; Zheng et al., 2021). However, the excessive amount of ROS accumulated at the injury site hampers the wound healing process. Consequently, to decrease the ROS level in the wounds, many ROS-scavenging materials have been incorporated into hydrogel dressings, such as antioxidants, enzymes and nanomaterials (Cheng et al., 2021). Antioxidants can be either directly loaded into hydrogels or conjugated with the polymer chains to achieve their localized and sustained release (Ren et al., 2021). These types of hydrogels were able to eliminate excess ROS production. For example, TA, a natural plant-derived polyphenol, has attracted widespread interest because of its outstanding biological functions such as antioxidative, antibacterial and anti-inflammatory properties. Inspired by these characteristics, TA was introduced into multifunctional hydrogels for infected wound healing application (He et al., 2020; Ma et al., 2020). Similarly, the antioxidant gallic acid was conjugated onto gelatin chains and blended with gelatin-hydroxyphenyl propionic (GH) hydrogels to fabricate an injectable hydrogel (Le Thi et al., 2020). Because of their ROS-scavenging potential, these hydrogels effectively reduced intracellular ROS production and accelerated wound closure. Interestingly, the ROS-scavenging hydrogels can also be developed by using a ROS-responsive linker. In the presence of ROS-sensitive linkers, such hydrogels were able to consume excessive ROS and induce drug release to inhibit bacterial infection, modulate inflammatory response, and promote angiogenesis and wound healing (Zhao et al., 2020; Yang et al., 2021; An et al., 2022). Since the oxygen acts as a key factor in maintaining normal cellular metabolism and promoting cell proliferation, to improve the healing process, a feasible strategy involves the simultaneous elimination of ROS and concomitant supply of oxygen at the wound site. Liu et al. designed an injectable hyaluronic acid (HA)-based hydrogels consisting of manganese dioxide (MnO_2_) nanosheets, M2 macrophages-derived exosomes and FGF-2 (Xiong et al., 2021). The MnO_2_ nanosheets can efficiently catalyze the decomposition of the excess ROS (H_2_O_2_) into oxygen (O_2_), thus reducing the ROS levels and increasing O_2_ production to provide cellular protective action against oxidative microenvironment. The *in vivo* results manifested that these hydrogels can create a favorable microenvironment to accelerate healing of diabetic wounds by alleviating oxidative stress and inducing angiogenesis.

The topography of biomaterials is an important biophysical cue which affects cellular behavior, such as adhesion, proliferation and differentiation (Chaudhuri et al., 2020). Nanofibrous materials which exhibit similar microstructure to the ECM, are considered to be ideal candidates for wound dressing (Chen et al., 2019). Electrospinning technology has been widely used for fabricating nanofibrous wound dressings. The Spirulina extract was loaded into electrospun PCL nanofibers to reduce ROS production and restore cellular activities in a cutaneous wound healing model (Jung et al., 2016). The Spirulina extract-loaded PCL scaffolds exerted significant antioxidant and anti-inflammatory activities to induce wound repair. Similarly, diabetic patients, the excessive ROS and inflammatory factors delay wound healing. To address this issue, Lin et al. fabricated hierarchical micro/nanofibrous scaffolds by using electrospinning and crystal engineering methods to achieve the sustained release of curcumin and Zn^2+^, which acted synergistically and not only reduced ROS level but also impeded the secretion of inflammatory factors (Wang Y. et al., 2020). The *in vivo* experiments further demonstrated a reduction in the oxidative stress and anti-inflammatory response, whereas an enhancement in ECs proliferation and neo-angiogenesis, ultimately translating into enhanced diabetic wound healing. Li and co-workers developed redox-sensitive poly (N-isopropylacrylamide-acrylic acid) (PNA) nanogels-incorporated nanofibrous poly (l-lactic acid) membranes (Zhang S. et al., 2021). The disulfide-containing PNA nanogels can balance the ROS level to create favorable microenvironment for tissue repair. Consequently, the PNA-containing nanofibrous membranes accelerated wound healing process via adjusting the ROS level.

Bacterial-infected wounds could not heal quickly (Ji et al., 2021; Wang et al., 2021). Nanomaterials exhibiting light-responsive multifunctional properties were designed as an enticing platform for the management of bacterial-infected wounds. To leverage synergistic chemical and photodynamic therapy for bacteria-contaminated skin wounds, Wang et al. developed photosensitizer chlorin e6 (Ce6) and magnesium (Mg)-contained nanocomplexes (Hu et al., 2019). The multifunctional nanoparticles could efficiently produce ROS under laser irradiation to kill the bacteria. Additionally, ROS-responsive release of Mg^2+^ from the nanoparticles could promote cell proliferation and migration and significantly accelerate wound healing. Xia et al. synthesized ROS-responsive poly-(1,4-phenyleneacetone dimethylene thioketal) (PPADT) nanoparticles loaded with stromal cell-derived factor-1α (SDF-1α), which can achieve targeted delivery of SDF-1α to wound sites with high concentrations of ROS (Tang et al., 2015). The thioketal bonds in the PPADT can be cleaved in the presence of ROS, which then lead to the local release of SDF-1α to induce bone marrow mesenchymal stem cells (BMSCs) homing and wound healing. Since surgical sutures are often used for wound closure, ROS-scavenging gallic acid-based nanoparticles (GANPs) coated collagen sutures were fabricated (Zhu et al., 2021). These sutures with GANPs coating can effectively scavenge ROS, promote macrophages polarization toward M2 phenotype, upregulate the expression level of anti-inflammatory factors, and accelerate wound healing process.

Microneedle patches are novel therapeutic systems in the field of wound healing. They have minimal invasion towards the skin and imperceptible pain after treatment. To achieve ROS-responsive on-demand drug delivery in the inflammatory tissues, a drug-loaded ROS-responsive poly (vinyl alcohol) (RR-PVA) microneedle patch was prepared (Zhang et al., 2018). A dual phenylboronic acid contained linker was introduced into PVA matrix, which can be cleaved by the ROS. With the ROS-dependent degradation of RR-PVA, sustained release of antibiotic clindamycin (CDM) was achieved. Thus, the CDM-loaded microneedle patch can deliver drugs into the dermis for acne vulgaris treatment. Therefore, it is a feasible and efficient strategy to develop ROS-responsive biomaterials to manipulate the balance of ROS levels for wound healing applications.

## Conclusions and Outlook

ROS play a significant role in numerous biological functions. A lack or an excess of oxidative stress and ROS may cause different types of diseases, including auto-immune diseases, cardiovascular diseases, and neurodegenerative diseases and so on. The excessive ROS and oxidative stress can induce strong inflammatory response to interfere tissue repair as well as perturb tissue repair and disturb immune system. The implantation of biomaterials also elevates oxidative stress as well as recruit inflammatory cell types. Overall, this necessitates a thorough understanding of ROS-mediated cellular/tissues injury and devise strategies to manage ROS level. A further insight on ROS signaling in injury and tissue microenvironment is warranted.

So far, a myriad of ROS scavengers, including systematically delivered nanoparticles, therapeutics, and biomaterials have been put forwarded. It is however only the latter which can help realize ROS regulation, as well as ROS-responsive payload release for inflammation regulation and tissue repair. Biomaterial with the inherent ability to regulate ROS by scavenging ROS have been designed, including those containing ROS-responsive linkers, radical scavenging polymers, controlled release of biologics or therapeutics, antioxidants, enzymes, and nanomaterials and have been used for the regeneration of infarcted heart, injured intervertebral disk, defected bone/cartilage tissues and skeletal muscles, chronic diabetic wounds, and cell therapy. ROS-responsive biomaterials which can help release biological signaling molecules, growth factors, and therapeutics in injury microenvironment and cell-mediated manner could help regulate ROS as well as alleviate oxidative stress and modulate tissue repair. These ROS-regulating biomaterials need further attention of the scientific community. Since elevated oxidative stress can perturb tissue repair and cellular functions, biomaterials with the ability to concurrently regulate ROS, alleviate oxidative stress, induce tissue regeneration, and enhance stem cell function, including survival, retention, and engraftment hold great promise for regenerative medicine and tissue regeneration and need further attention.

Tissue regeneration involves multiple steps manifesting the key role of inflammatory and fibrotic responses, intelligent biomaterials with multiple functionalities and with the capabilities to regulate ROS, and modulate macrophages polarization towards M2 phenotypes and induce tissue repair are warranted. The ROS-responsive biomaterials can be processed into different shapes and structures, such as nanoparticles, nanofibers, microneedles, hydrogels, and so on to further enhance their applicability and harness ROS for tissue regeneration.
